# Independent Review Organization and Proton Therapy: Multistate Analysis and Legal Procedural Strategies

**DOI:** 10.1016/j.ijpt.2025.100741

**Published:** 2025-02-17

**Authors:** Eric D. Brooks, Terence T. Sio, Matthew S. Ning, Christopher G. Morris, Nancy P. Mendenhall, Montreal Turner, Noreen K. Vergara, Matthew Palmer, Mark E. Artz

**Affiliations:** a Premier Radiation Oncology Associates, Clearwater, Florida, USA; b Department of Radiation Oncology, Mayo Clinic, Phoenix, Arizona, USA; c Department of Radiation Oncology, MD Anderson Cancer Center, Houston, Texas, USA; d University of Florida Health Proton Therapy Institute, University of Florida College of Medicine, Jacksonville, Florida, USA; e Legion Healthcare Partners, LLC, Houston, Texas, USA; f Husch Blackwell LLP, Kansas City, Missouri, USA

**Keywords:** Utilization review, Medical necessity review, Appeal(s), Medical necessity criteria, Adverse benefit decision

## Abstract

**Purpose:**

Securing insurance authorization for proton therapy remains a challenge for many centers. When health insurance or employer-sponsored health plans deny coverage, Independent Review Organizations (IROs) can review proton therapy cases. However, despite providing an independent review pathway, IROs are often underutilized in securing approvals for care following a denial.

**Materials and Methods:**

We analyzed trends in IRO approvals, strategies, and legal procedures using publicly available data from California (CA), Washington (WA), and New York (NY).

**Results:**

The aggregate analysis of the 3 states revealed an IRO average approval rate for proton therapy of 42.1%, with varying trends across states. All 3 states showed increases in IRO approval rates over time, averaging annual increases of +5.0%, +2.3%, and +7.2% for CA, WA, and NY, respectively. Sarcoma showed the highest IRO approval rate at 84.6%, followed by GYN cancers at 55.6% and breast cancer at 51.4%. CNS tumors and lymphomas had moderate approval rates at 44.7% and 40.0% respectively. Head and neck cancers had a 33.3% approval rate, while thoracic malignancies were at 36.8%. The lowest IRO approval rate was seen in prostate cancer at 16.5%. Qualitative analysis revealed that referencing guidelines, discussing published studies, citing trial inclusion, and submitting personalized letters were associated with higher IRO approval rates.

**Conclusion:**

IRO reviews provide a more objective remedy for patients denied care through internal appeals, particularly for plans with historically unfavorable proton policies. Our study demonstrates that IRO appeals provide a valuable pathway to proton therapy access with higher overturn rates improving significantly in recent years. Nearly half of initially denied patients eventually received approval through this process. Proton centers should strategically utilize IRO reviews to increase patient access and improve approval chances.

## Introduction

Securing insurance authorization for proton therapy remains challenging for many centers. Independent Review Organizations (IROs) offer a pathway for reviewing cases when health insurance or employer-sponsored plans deny proton payment, but they are underutilized in securing benefits after an internal denial. The goal was to provide insights into the IRO decision-making process for the proton therapy community and to generate hypotheses and strategies for improving access to proton therapy. To our knowledge, this is the first data analysis of its kind.

Federal and state laws regulate health plans and specify the process for submitting cases to an IRO for external review. As third-party reviewers, IROs either uphold or overturn internal adverse benefit determinations, and their decisions are legally binding on health plans to cover denied proton treatments.

IROs are underutilized due to a lack of awareness, unfamiliarity with applicable laws, inadequate processes for submission, and limited administrative resources at centers. However, strategically submitting to IROs can help demonstrate medical necessity to an independent body to improve access to appropriate proton therapy.

This report examines the IRO external review process and provides a multistate analysis of IRO appeal decisions for proton therapy, focusing on overturn rates by diagnosis, reasons for approvals and denials, and strategies to better engage with this underused option.

### Independent review organizations and common commercial insurance terminology

Following the implementation of the Affordable Care Act in 2010 IROs became a requirement for every state.^1^ IRO discussions primarily apply to commercial health insurance plans, as noncommercial or government payors use alternative dispute resolution methods for benefit denials. Within commercial insurance, we focus on 2 major types: fully-funded and self-funded plans, both typically employer-sponsored and most relevant to proton therapy patients since most Americans receive health coverage through employers.

IROs operate as third-party medical review entities that employ board-certified physicians across various specialties to evaluate medical necessity appeals, though they do not formally partner with proton centers or providers. The IRO physician reviewers, who must be actively practicing and have no conflicts of interest, make determinations based on their clinical expertise, current medical literature, and treatment guidelines while remaining independent from both the health care facility and insurance plan. The administrative staff at IROs manage case intake and documentation but do not influence medical necessity decisions, which are made solely by the physician reviewers who must be matched appropriately by specialty to the case being reviewed.^2^, ^3^

### When ndependent review organization applies: Background on fully-funded plan and self-funded plan

Fully-funded plans involve employers contracting with state-regulated insurers for employee health care coverage, transferring all risk to the insurer. These plans follow state laws and accreditation standards from organizations like the National Committee for Quality Assurance or Utilization Review Accreditation Commission, which regulate prior authorization (PA), appeals, and external review procedures.^1^, ^4^

Self-funded plans, regulated by the Federal Employee Retirement Security Act (ERISA) statute,^5^, ^6^ involve employers assuming financial risk and often contracting with Third-Party Administrators (TPAs) for benefits management and utilization review. These plans offer greater flexibility for negotiating care approval through employer engagement.^7^

### Prior authorization processing and tiers

All plans have response-time tiers for PA: routine (15-day), urgent (72 hours), and emergent (24 hours). For cancer cases, urgent reviews are appropriate given the threat to life. Centers should consistently request urgent reviews, as federal law prevents downgrading such requests when documented by clinicians. This ensures timely responses and preserves appeal options.

### General appeal process

Despite state-by-state variations, most plans follow timelines similar to ERISA standards. While TPAs often manage PA, plans retain ultimate responsibility for meeting timelines and following procedures. When initial submissions are denied, patients have legal rights to internal appeals, which must be resolved within 72 hours for urgent cases. Appeals should specifically address denial rationales and include both medical details and any procedural violations.

### Prepare for Independent Review Organization from the beginning

Early preparation for possible IRO review is crucial. Representatives should obtain Authorization of Representative forms during initial appeals to prevent delays. If the first appeal fails, assessing the success rates of second appeals can inform whether to proceed directly to IRO. Appeals should incorporate guideline deviations, authoritative support, and relevant literature, tailored to patient-specific contexts.^2^

### Navigating the Independent Review Organization process: Selection, timelines, and impact for self-funded and fully-funded plans

Federal law mandates external IRO availability for both self-funded and fully-funded plans.^8^, ^9^ IROs conduct independent reviews based on medical necessity and standard care, requiring specific documentation (Table 1) and following regulated timelines. Their decisions are binding and generally increase approval rates.^3^

**Table 1:** Necessary IRO documents.

Appeal letter
Medical records
Authorization of representative (AOR) form
Medical release consent forms
Comparison plan
Expedited request form signed by provider
Copy of health plan's final internal appeal denial letter (fully-funded only)
Copy of insurance card (fully-funded only)

**Abbreviation:** IRO, Independent Review Organization.

IRO determinations rely on clinical guidelines, peer-reviewed literature, and patient-specific records, with specialty-matched reviewers. While patients can challenge unfavorable decisions, courts rarely overturn IRO rulings except for egregious errors.^10^ Centers should understand relevant laws and use internal data to guide IRO strategy decisions.

Insurance plans are responsible for selecting IRO vendors but must do so on a rotating basis from state-approved organizations to ensure impartiality and prevent preferential selection. This structured approach to vendor selection, combined with the requirement for plans to cover review costs helps maintain the independence and fairness of the external review process.^3^ The insurance company or the Health and Human Services (HHS) funds the IRO appeals process. This cost is not passed onto the center or the patient.

The IRO vendor is based on whether it is fully-funded or self-funded. Fully-funded plans follow state guidelines and are assigned to state-approved external contractors. Self-funded plans are governed by ERISA and are sent to Federally approved contractors, that is, MAXIMUS.

For self-funded plans, the HHS-Administered Federal External Review Process (HHS-AFERP) is available at no cost to the health insurance plan, the consumer, or a consumer’s authorized representative. Issuers that elect to use the HHS-AFERP and consumers whose plan is participating in the HHS-AFERP, will work with the designated federal contractor, MAXIMUS Federal Services, Inc (MAXIMUS). For an expedited external review, the MAXIMUS examiner must provide notice of the final external review decision as expeditiously as the medical circumstances require and within 72 hours once the examiner receives the request for the external review. MAXIMUS must deliver the notice of the final external review decision to the claimant and the health insurance issuer as soon as possible.

The ACA mandates that health plans, including fully-funded plans, provide an external review process for claim denials. The cost of this external review is borne by the health insurance plan, not by the patient. This ensures that the review process is accessible without financial burden to the individual seeking the appeal. To receive an expedited external review with a fully-funded plan, the TPA and health plan must provide notice of the final external review decision as expeditiously as the medical circumstances require and within 72 hours once the examiner receives the request for the external review. The external reviewer must deliver the notice of the final external review decision to the claimant and the health insurance issuer as soon as possible, per federal regulations under the Affordable Care Act and as outlined in the Summary Plan Description (SPD).

## Methods

Publicly available IRO decision data from the Departments of Insurance in 3 US states with existing proton therapy centers were analyzed. Each state published data in different formats, with varying data points, but all included reviewer responses and IRO decisions for proton therapy patients. Our analysis included available proton IRO claims from 2014 to 2024, 2016 to 2024, and 2019 to 2024, in the states of California (CA), Washington (WA), and New York (NY).^11^, ^12^, ^13^

**Figure 1:**
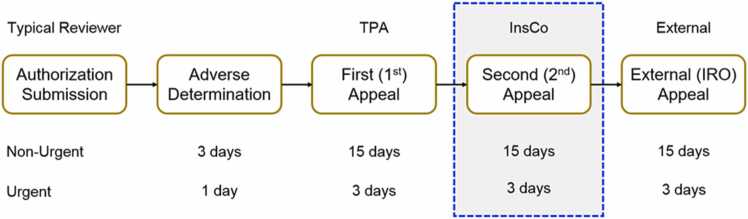
General appeals process for Nonurgent and Urgent insurance plan and IRO appeals. Patients with both fully and self-funded plans can reach the IRO level of appeal only after exhausting internal appeals or having the option to skip in an event such as a timeline violation or state code allows. Ensuring the plan understands the need for approval at the urgent tier timeline is critical for patients with cancer because timely delivery of care is required for best clinical outcomes. Because the total time to reach IRO can take up to 33 days with a routine tier submission, but only 7 to 10 days with an urgent tier designation, triggering the urgent tier timeline is often crucial for patients. During the authorization process, the plan will determine the level of review at the beginning of the PA process, and setting the timeline at the urgent tier is often required at the time of submission which better aligns the subsequent steps of the utilization review process to ensure patients stand the best odds of achieving appropriate and timely access to care. “Urgent” should be denoted in provider notes to ensure insurance companies, plans, other payors, and TPAs process such requests for authorization as urgent.

IRO approval rates by insurance plan and individual IRO reviewer organization were analyzed for the state of New York due to it having a relatively large number of IRO cases and available data. Washington provided similar data but there were only 33 versus 131 cases in New York. The California database did not provide data on the insurance plan type or IRO organization. These 3 states were chosen based on the availability of public databases at the time we began the study. The PA timeline, including the IRO option, is outlined in Figure 1.

Qualitative analysis focused on common reasons for approval or denial and identified trends among IRO reviewers. Based on these analyses, we deduced and inferred strategies to improve IRO overturns of health plan denials. The “average IRO response time” for proton therapy cases, as reported in our analysis, refers to calendar days rather than business days. The response times are calculated from the date of receipt to the date of determination without excluding weekends or holidays.^11^, ^12^, ^13^

Statistical analyses were performed using Python (version 3.12) with SciPy (version 1.11.3) for statistical testing, NumPy (version 1.24.3) for numerical computations, statsmodels (version 0.14.0) for regression modeling, and Matplotlib (version 3.8.0) for data visualization.^14^, ^15^, ^16^, ^17^ Spearman rank correlation tests were used to assess trends over time, and logarithmic regression analyses were performed using SciPy's stats module. To evaluate differences in approval trends between states, approval was treated as a binary endpoint and assessed as a function of both time and state using logistic regression models. Interaction terms between time and state were included to evaluate whether the change in approval rates over time differed significantly between states. A significant interaction term would indicate that the trajectory of approval rates over time differed between the compared states. Statistical significance was defined as *P* < .05.

## Results

**Figure 2:**
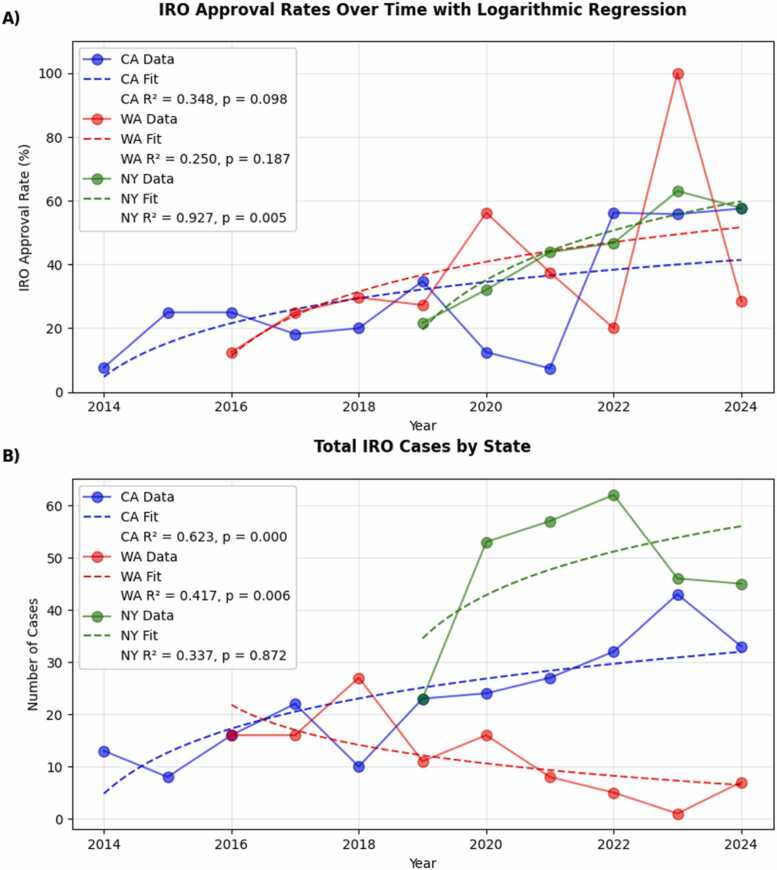
IRO approval rate by state (A). Total IRO cases by state (B). (A) The IRO approval rate by year for California (CA), Washington (WA), and New York (NY). All 3 states show positive trends in IRO approval rates over time, with average annual increases of +5.0%, +2.3%, and +7.2% for CA, WA, and NY, respectively. The IRO approval rate increased at a statistically significant rate only for NY (*P* = .002), while CA (*P* = .094) and WA (*P* = .182) showed nonsignificant positive trends. (B) The total number of IRO cases increased significantly in CA (*P* = .003) and NY (*P* = .008), with average annual increases of 2.2 and 4.4 cases, respectively, growing from 23 cases in 2019 to peaks of 43 cases (2023) in CA and 62 cases (2022) in NY. Despite comparable approval rates to the other states, WA showed a significant declining trend in total cases (*P* = .038), decreasing by approximately 1.3 cases annually, with a notable drop to one case in 2023 before rebounding to 7 cases in 2024.

The aggregate analysis of the 3 states revealed an IRO average approval rate for proton therapy of 42.1%, meaning IROs allowed almost half of patients to receive proton therapy who otherwise would not have. All 3 states showed an increase in overturn rates of internal denials through use of IRO, averaging +5.0%, +2.3%, and +7.2% per year in aggregate for CA, WA, and NY, respectively (Figure 2A). The increase in CA's approval rates showed a trend but was not statistically significant (*P* = .094), NY shows the strongest and most significant trend (*P* = .002), while WA shows a positive trend that was not statistically significant (*P* = .182).

Analysis of IRO case volumes revealed contrasting patterns across states (Figure 2B). California demonstrated a statistically significant increase in case volumes (*P* = .003) with a steady growth of 2.2 cases per year. Washington showed a significant decline in cases over time (*P* = .038), with volumes decreasing by approximately 1.3 cases annually. In contrast, New York exhibited the strongest positive trend (*P* = .008) with a substantial increase of 4.4 cases per year on average. This varying pattern suggests regional differences in IRO utilization, with California and New York demonstrating greater adoption of the IRO process despite starting from different baseline volumes. The declining trend of total IRO cases in Washington, despite improved approval rates, may be related to the change in ownership of the Seattle Proton Therapy Center in 2021, the Seattle Proton Therapy Center previously operated independently but is now part of a larger cancer center and may have changed its use of IROs for proton therapy insurance approval.^18^

Logistic regression analysis examined differences in how approval rates changed over time between states. The interaction between time and state was not significant when comparing NY to CA (*P* = .474), suggesting that despite NY's steeper improvement (+7.2% per year) compared to CA's more modest increase (+5.0% per year), their trajectories were not statistically distinct. Similarly, the interactions between CA and WA (*P* = .350) and between NY and WA (*P* = .170) were not significant, indicating that despite different annual increases (+5.0%, +7.2%, and +2.3% for CA, NY, and WA respectively), the trajectories of approval rates over time were not statistically distinguishable between any pair of states. However, the time coefficient was significant in the CA versus NY comparison (*P* < .001), confirming an overall positive trend in approval rates over time when considering these states together.

**Figure 3:**
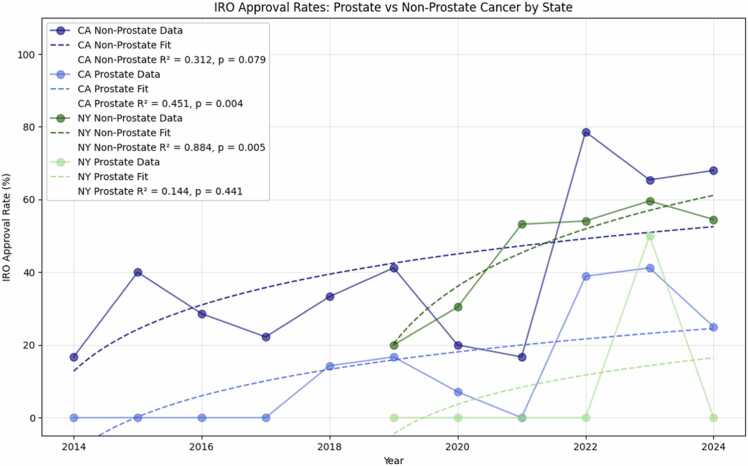
IRO Approval Rates by Cancer Type and State. Analysis of IRO approval rates revealed distinct patterns between prostate and nonprostate cases in both California and New York. Nonprostate cases consistently demonstrated higher approval rates and more favorable trends in both states. In CA, nonprostate cases showed a significant increase (*P* = .018, +5.1% annually) reaching 68.0% by 2024, while prostate cases, despite recent improvement to 38.9% to 41.2% in 2022-2023, showed a nonsignificant trend (*P* = .064, +2.5% annually) and decreased to 25.0% in 2024. Similarly, in NY, nonprostate cases showed strong, consistent improvement (*P* = .004, +6.9% annually), reaching 54.5% by 2024, while prostate cases remained predominantly at 0% approval, except for a brief increase to 50.0% in 2023 (*P* = .872). This divergence in approval patterns between cancer types was particularly pronounced in NY, where prostate cases showed minimal improvement over time, and in CA where prostate approvals, though improving, remained substantially lower than nonprostate cases.

Analysis of IRO approval rates revealed distinct patterns between prostate and nonprostate cases in both California and New York (Figure 3). Nonprostate cases consistently demonstrated higher approval rates and more favorable trends in both states. In CA, nonprostate cases showed a significant increase (*P* = .018, +5.1% annually) reaching 68.0% by 2024, while prostate cases, despite recent improvement to 38.9% to 41.2% in 2022-2023, showed a nonsignificant trend (*P* = .064, +2.5% annually) and decreased to 25.0% in 2024. Similarly, in NY, nonprostate cases showed strong, consistent improvement (*P* = .004, +6.9% annually), reaching 54.5% by 2024, while prostate cases remained predominantly at 0% approval, except for a brief increase to 50.0% in 2023 (*P* = .872). This divergence in approval patterns between cancer types was particularly pronounced in NY, where prostate cases showed minimal improvement over time, and in CA where prostate approvals, though improving, remained substantially lower than nonprostate cases.

By histology, sarcoma showed the highest IRO approval rate at 84.6% (11 overturned vs 2 upheld), followed by GYN cancers at 55.6% (5 vs 4) and breast cancer at 51.4% (71 vs 67) (Table 2). CNS tumors and lymphomas had moderate approval rates at 44.7% (34 vs 42) and 40.0% (12 vs 18), respectively. Head and neck cancers had a 33.3% approval rate (32 vs 64), while thoracic malignancies were at 36.8% (14 vs 24). The lowest IRO approval rates were seen for GI cancers at 20.0% (4 vs 16) and prostate cancer at 16.5% (22 vs 111). These results indicate substantial variation in IRO approval rates across different cancer types.

**Table 2:** IRO overturn approval rate by histology by state

Disease site	CA	NY	WA	CA OT%	WA OT%	NY OT%	Total OT	Total upheld	% of total	% OT
Breast	19	45	7	43.2%	46.7%	57.0%	71	67	21.1%	51.4%
CNS	9	19	6	52.9%	23.1%	57.6%	34	42	11.6%	44.7%
Esophagus	0	2	0	0.0%	0.0%	100.0%	2	4	0.9%	33.3%
GI	0	4	0	0.0%	0.0%	21.1%	4	16	3.1%	20.0%
GYN	2	3	0	100.0%	0.0%	42.9%	5	4	1.4%	55.6%
H&N	3	24	5	20.0%	35.7%	35.8%	32	64	14.7%	33.3%
Lymphoma	5	5	2	50.0%	28.6%	38.5%	12	18	4.6%	40.0%
Prostate	19	3	0	18.8%	0.0%	9.7%	22	111	20.3%	16.5%
Sarcoma	4	7	0	80.0%	0.0%	87.5%	11	2	2.0%	84.6%
Thoracic	3	9	2	33.3%	40.0%	37.5%	14	24	5.8%	36.8%
Other	23	10	11	51.1%	29.7%	76.9%	44	51	14.5%	46.3%

Total	87	131	33	34.7%	30.8%	44.3%	251	403	100.0%	42.1%

**Abbreviations:** IRO, Independent Review Organization; OT, overturn; WA, Washington; NY, New York; CA, California; H&N, head and neck.

Analysis of New York-specific insurance plan data revealed notable patterns in Independent Review Organization (IRO) approval rates (Table 3). Medicaid cases showed strong approval rates at 50.5% (47 overturned vs 46 upheld) while comprising 31.4% of all cases. Among private insurers, indemnity plans and Preferred Provider Organization (PPO) plans demonstrated moderate success with approval rates of 48.6% and 43.3%, respectively, while Health Maintence Organization (HMO) plans had a lower rate of 38.3%. Among commercial insurers, Aetna achieved a 100% approval rate, though on a small sample of 4 cases.

**Table 3:** IRO approval by insurance plan and IRO organization for proton therapy—New York

IRO approval by insurance plan					
Plan type	Overturned	Upheld	Total	% of total	% overturned
Medicaid	47	46	93	31.4%	50.5%
HMO	23	37	60	20.3%	38.3%
Indemnity	17	18	35	11.8%	48.6%
EPO	13	27	40	13.5%	32.5%
PPO	13	17	30	10.1%	43.3%
Fidelis care	6	3	9	3.0%	66.7%
Aetna commercial	4	0	4	1.4%	100.0%
Oxford commercial	3	5	8	2.7%	37.5%
Essential plan	2	9	11	3.7%	18.2%
Self-funded	2	2	4	1.4%	50.0%
Excellus	1	1	2	0.7%	50.0%

**Total**	**131**	**165**	**296**	**100%**	**44.3%**


**IRO approval by reviewer organization**					
**IRO organization**	**Overturned**	**Upheld**	**Total**	**% of total**	**% overturned**
					
**Acentra health/Kepro/IMEDECS**	**37**	**100**	**137**	**46.3%**	**27.0%**
2019	0	7	7		0.0%
2020	2	22	24		8.3%
2021	2	21	23		8.7%
2022	14	27	41		34.1%
2023	12	11	23		52.2%
2024	7	12	19		36.8%

**IPRO**	**54**	**37**	**91**	**30.7%**	**59.3%**

2019	0	7	7		0.0%
2020	10	6	16		62.5%
2021	17	3	20		85.0%
2022	9	3	12		75.0%
2023	9	9	18		50.0%
2024	9	9	18		50.0%

**MCMC, LLC**	**40**	**28**	**68**	**23.0%**	**58.8%**

2019	4	4	8		50.0%
2020	2	7	9		22.2%
2021	6	6	12		50.0%
2022	10	8	18		55.6%
2023	10	2	12		83.3%
2024	8	1	9		88.9%

**Total**	**131**	**165**	**296**		**44.3%**

**Abbreviation:** IRO, Independent Review Organization. PPO, Preferred Provider Organization. EPO, Exclusive Provider Organizations. HMO, Health Maintence Organization.

The analysis of reviewer organizations reveals distinctive patterns in approval rates across different IRO reviewer organizations during the 2019-2024 period. MCMC, LLC demonstrated relatively high IRO approval rates, with approval rates consistently improving year over year, reaching a peak of 88.9% in 2024. IPRO similarly also showed a high rate of approvals, maintaining approval rates above 50% throughout most of this period. IMEDECS approval rates were significantly lower and averaged only 27.0%, although their year-over-year performance did show gradual enhancement, particularly after 2020 with a peak of 52.2% in 2023 before returning to 36.8% in 2024.

For nonproton radiation therapy cases in New York (Table 4), the overall approval rate was notably higher at 73% (90 overturned vs 34 upheld), with consistent improvement from 50% in 2019 to 77% in 2023. Among specific radiation modalities, intensity modulated radiation therapy showed strong success with a 79% approval rate (26 vs 7), while newer technologies like Y90 and selective internal radiation therapy achieved 86% (12 vs 2) and 80% (4 vs 1) approval rates respectively. Several other modalities, including photochemotherapy, brachytherapy, and various other radiation procedures, achieved 100% approval rates, albeit with smaller sample sizes. This pattern suggests that IRO reviewers may be more amenable to approving established radiation therapy technologies compared to proton therapy, though the smaller sample sizes for some modalities may limit the ability to draw inferences from interpreting these results.

**Table 4:** IRO Approval for nonproton radiation procedures—New York.

Radiation therapy (nonproton)				
Year	Overturned	Upheld	Total	% overturned
2019	2	2	4	50%
2020	16	6	22	73%
2021	18	7	25	72%
2022	13	5	18	72%
2023	23	7	30	77%
2024	18	7	25	72%

**Total**	**90**	**34**	**124**	**73%**


Radiation therapy procedures (nonproton)				
Year	Overturned	Upheld	Total	% overturned
				
SBRT	22	12	34	65%
IMRT	26	7	33	79%
RT	9	6	15	60%
Y90	12	2	14	86%
SRS	5	2	7	71%
SIRT	4	1	5	80%
Optune	2	3	5	40%
Hydrogel Spacer	2	1	3	67%
Photochemotherapy	2	0	2	100%
Brachytherapy	1	0	1	100%
Phototherapy	1	0	1	100%
UV	1	0	1	100%
Laser	1	0	1	100%
MRI	1	0	1	100%
Other	1	0	1	100%

**Total**	**90**	**34**	**124**	**73%**

**Abbreviations:** IRO, Independent Review Organization; SBRT, stereotactic body radiation therapy; IMRT, intensity modulated radiation therapy; RT, radiation therapy; Y90, Yttrium-90; SRS, stereotactic radio surgery; SIRT, internal radiation therapy; UV, ultra violet; MRI, magnetic resonance imaging.

Regarding time to determination, CA had an average IRO response time of 5.8 calendar days for expedited/urgent IRO submissions and 20.9 days for standard cases, with an overall average of 8.2 days. In WA, the average IRO response time of 3.2 days was reported for expedited/urgent IRO submissions and 17.7 days for standard cases with an average overall IRO response time of 16.2 days. Determination time data was unavailable for NY.

## Discussion

Insurance authorization for proton therapy represents a significant challenge in radiation oncology, with IROs offering a powerful yet underutilized pathway to improve patient access. Studies demonstrate that IRO review can result in a significant increase in patient access, a favorable outcome for both patients and providers.^2^, ^3^ Understanding the role and impact of IROs requires examining them within the broader context of proton therapy insurance approvals.

The existing literature highlights substantial disparities between commercial and public insurance coverage. Studies show initial approval rates for commercial insurance ranging from only 30% to 32.5% compared to Medicare's 91% to 97.4%.^19^, ^20^ When patients appeal initial denials, success rates vary considerably, with some studies reporting 68% success on appeal, though this typically delays treatment by 3 weeks or more.^19^ Of particular concern, research indicates that 19% of denied patients ultimately abandon radiation treatment altogether.^21^ Young adult patients face unique challenges, with significantly lower initial approval rates (48%) compared to pediatric patients (99%).^22^

Our comprehensive analysis demonstrates that external review through IROs provides an increasingly viable pathway for proton therapy access to denied patients, with an aggregate approval rate of 42.1%. This success rate becomes even more significant when considering disease-specific outcomes, with some combinations of diagnoses and reviewer organizations achieving approval rates approaching 90% in recent years.

Our analysis also indicates that IRO approvals have increased over time, likely reflecting the expanded indications for proton therapy by national guidelines such as American Society for Radiation Oncology (ASTRO) and the National Comprehensive Cancer Network. The 2023 ASTRO Proton Model Policy expansion of recommended indications and the lobbying work leading up to it appears to correlate with increased approval rates, particularly from the 2021 to 2024 time period analyzed.^23^ However, a troubling trend emerged when reviewers justified denials by citing that the Plan acted with "sound medical judgment," reflecting nonmedical entities influencing care decisions. This raises concerns, as many state laws restrict medical decision-making to licensed professionals and that IROs should evaluate cases de novo.

Moreover, the variation in approval rates among different IRO organizations is broad (eg, a range of 27% to over 80%) suggesting distinct differences in review methodologies. Improvements in objective review at the IRO level would likely make such variability narrower. Our qualitative analysis helped to shed light on where this variability arises. On analysis, many IRO decisions can appear subjective, as evidenced by multireviewer panels reaching different conclusions based on the same data (eg, 1 reviewer approving proton therapy based on DVH benefits, while another denies it for not exceeding precise Radiation Therapy Oncology Group/Quantitative Analysis of Normal Tissue Effects in the Clinic thresholds). Moreover, some approved proton therapy for trial patients, citing ASTRO or other national guidelines, while others denied it as experimental. In cases with published data, some reviewers approved based on the evidence, while others denied due to the absence of randomized controlled trials. Therefore, work on improving consistency and objectivity at the IRO level is still needed, despite the benefit of using this less subjective pathway.

Overall, our findings suggest several strategic priorities for proton centers. Organizations should develop robust systems for identifying and expediting high-probability cases, particularly for diagnoses showing strong approval trends in their state. Success factors in IRO approval include comprehensive documentation of patient-specific elements, clear guideline references, and detailed dosimetric comparisons at time of submission (Table 5). However, the proton center team must also be able to navigate timeline pressures, documentation requirements, and the need for specialized knowledge of state-specific regulations to be optimally successful at IRO. Doing so can mean an additional 1 in 2 patients receiving proton therapy who otherwise would not have received it.

**Table 5:** IRO differences in approvals and denials with author recommendations.

Reviews who approve	Reviewers who deny	Author recommendations
Patient-specific factors cited	Lack of patient-specific factors included	Include as many unique patient-specific factors as possible to substantiate (need to review and reference the PMH, PSH, Meds, SH, FH, etc.—conspicuous and inconspicuous details that could make protons especially critical)
National guidelines support (directly or indirectly)	National guidelines do not expressly support	Reference how the case relates to guideline endorsement, directly or indirectly; NOTE patients on trial can fall under endorsed guidelines, including possibly registry; consider stating that CMS covers the entity, if applicable (demonstrated case is supported by the government guidelines)
Published data support	No published data, nonrandomized, wavering conclusions	Published data to be included in all cases, even if indirect publications need to be used. Then, discuss how the data apply to the patient specifically
Dosimetry benefit with a comparison plan	Dosimetry Benefit does not exceed QUANTEC/RTOG	Highlight any/all QUANTEC/RTOG thresholds that are exceeded; label plan as unsafe w/o protons

**Abbreviations:** IRO, Independent Review Organization; PMH, past medical history; PSH, past surgical history; Meds, medications; SH, social history; FH, family history; QUANTEC, Quantitative Analysis of Normal Tissue Effects in the Clinic; RTOG, Radiation Therapy Oncology Group.

This study's limitations include the restricted data set from only 3 states and the relatively short 5 to 10 years of IRO experience examined. Additionally, changes in proton center ownership structures and COVID-19 impacts may have influenced recent trends.^24^ Despite these limitations, this analysis provides valuable first insights into the IRO process and its evolution.

## Conclusion

The consistent improvement in approval rates observed across states suggests that continued engagement with the IRO process, combined with strategic preparation and documentation, can help overcome historical barriers to proton therapy access. As this treatment modality continues to gain acceptance, centers that effectively leverage these insights position themselves to better serve their patients while potentially influencing broader acceptance of proton therapy in clinical practice. Future research should examine additional states' experiences with IRO processes and evaluate the long-term impact of recent guideline changes on approval patterns.

## Ethical approval

This project did not require IRB approval.

## Funding and Support

This study received no funding.

## Disclosure of the Use of Generative AI

During the preparation of this work the authors used Anthropic PBC to edit certain sections of the paper. After using this tool/service, the authors reviewed and edited the content as needed and take full responsibility for the content of the publication.

## Data Sharing Statement

The authors agree to share anonymized data upon reasonable request by researchers.

## Declaration of Competing Interest

The authors declare that they have no known competing financial interests or personal relationships that could have appeared to influence the work reported in this paper.
