# Supplementary material for: Independent Review Organization and Proton Therapy: Multistate Analysis and Legal Procedural Strategies
Source: Int J Part Ther. 2025 Feb 17;15:100741. doi: 10.1016/j.ijpt.2025.100741 (PMC11905844; doi:10.1016/j.ijpt.2025.100741)
Supplement: Supplementary file 1 — Supplementary material [file mmc1.docx]

| **Supplemental Table 1. Laws and Regulations Governing Internal and External Appeals by State [1,2]** | | | | | | | |
| --- | --- | --- | --- | --- | --- | --- | --- |
|  | **External Review (IRO)** | | | | | **Internal Review (Appeals)** | |
|  | Laws and Accreditation Standards Regulating Plans by State | | | | | | |
|  | **Fully-Funded** | | | | **Self-Funded** | **Fully-Funded** | **Self-Funded** |
|  | NAIC Member | | |  |  |  |  |
|  | Model Adoption | Related State Activity  (Meets Parallel or Similar) | HHS Administered Process | IRO Expedited Review Requirements Imposed on Payors Similar to ERISA (e.g., 72h)? |  | Internal Appeals Expedited Review Requirements Imposed on Payors Similar to ERISA (e.g., 72h)? |  |
| Alabama |  | Bulletin 9-23-2010. | HHS | Yes. Uniform Health Carrier External Review Model Act; Section 9(E)(1) | ERISA | Yes. NAIC Utilization Review and Benefit Determination Model Act; Section 10(B)(1)(a) | ERISA |
| Alaska | ALASKA ADMIN CODE tit 3, §§ 28.900 to 28.918 (2018) | Alaska Stat. Ann. §21.07.005 (2016); Bulletin 2018-5 (2018) |  | Yes. Uniform Health Carrier External Review Model Act; Section 9(E)(1) | ERISA | Yes. NAIC Utilization Review and Benefit Determination Model Act; Section 10(B)(1)(a) | ERISA |
| American Samoa | NO CURRENT ACTIVITY |  |  | Yes. Uniform Health Carrier External Review Model Act; Section 9(E)(1) | ERISA | Yes. NAIC Utilization Review and Benefit Determination Model Act; Section 10(B)(1)(a) | ERISA |
| Arizona |  | ARIZ REV STAT ANN §§ 20-2501 to 20-2511 (1993/2014); §§20-2537 to 02-2539 (1997/2013); Bulletin 2011-7 |  | Yes. Uniform Health Carrier External Review Model Act; Section 9(E)(1) | ERISA | Yes. NAIC Utilization Review and Benefit Determination Model Act; Section 10(B)(1)(a) | ERISA |
| Arkansas | 054.00.76 ARK. CODE R. §§ 1 to 19; Apps. A to D (2011/2012). | ARK CODE ANN §§ 20-9-901 to 20-9-914 (1989/2019); Bulletin 10-2011 (2011). |  | Yes. Uniform Health Carrier External Review Model Act; Section 9(E)(1) | ERISA | Yes. NAIC Utilization Review and Benefit Determination Model Act; Section 10(B)(1)(a) | ERISA |
| California |  | CAL HEALTH & SAFETY CODE § 1363.5 (1999/2000); § 1367.01 (2000/2010); §1370.4 (1997/2001); CAL. INS. CODE § 10145.3 (1996/2001); CAL. INS. CODE §§ 10169 to 10169.5 (1999/2015); NOTICE 5-17-2011 (2011). |  | Yes. Uniform Health Carrier External Review Model Act; Section 9(E)(1) | ERISA | Yes. NAIC Utilization Review and Benefit Determination Model Act; Section 10(B)(1)(a) | ERISA |
| Colorado |  | 3 COLO CODE REGS § 702-4:4-2-14; §702-4:4-2-21 (1997/2010) (previous model);; COLO REV STAT §§ 10-16-112 to 10-16-113 (1993/2019); § 104-115 (1993/2003); BULLETIN B-4.20 (REVISED #4) (2017) |  | Yes. Uniform Health Carrier External Review Model Act; Section 9(E)(1) | ERISA | Yes. NAIC Utilization Review and Benefit Determination Model Act; Section 10(B)(1)(a) | ERISA |
| Connecticut |  | CONN GEN STAT §§ 38a-591 to 38a-591n (2011/2019); CONN AGENCIES REGS § 38a-591 (2012/2017); Bulletin HC-74 (2009); Bulletin HC-84 (2011); Bulletin HC-93 (2013) |  | Yes. Uniform Health Carrier External Review Model Act; Section 9(E)(1) | ERISA | Yes. NAIC Utilization Review and Benefit Determination Model Act; Section 10(B)(1)(a) | ERISA |
| Delaware |  | 18 DEL ADMIN CODES §§ 1301-1.0 to 1301-14.0 (2007/2018); DEL. CODE ANN. tit.18, § 332  (1996/2012). |  | Yes. Uniform Health Carrier External Review Model Act; Section 9(E)(1) | ERISA | Yes. NAIC Utilization Review and Benefit Determination Model Act; Section 10(B)(1)(a) | ERISA |
| District of Columbia |  | D.C. CODE §§ 44-301.01 to 44-301.11 (1998/2012). |  | Yes. Uniform Health Carrier External Review Model Act; Section 9(E)(1) | ERISA | Yes. NAIC Utilization Review and Benefit Determination Model Act; Section 10(B)(1)(a) | ERISA |
| Florida |  | FLA. STAT. § 641.312 (2012/2018); MEMORANDUM 2010-006 (2010); MEMORANDUM 2011-07M (2011). | HHS | Yes. Uniform Health Carrier External Review Model Act; Section 9(E)(1) | ERISA | Yes. NAIC Utilization Review and Benefit Determination Model Act; Section 10(B)(1)(a) | ERISA |
| Georgia |  | GA CODE ANN §§ 36-46-1 to 33-46-14 (1990-1996); GA COMP R & REGS 120-2-58 (1196/2002); 120-2-20 (1998-2002) | HHS | Yes. Uniform Health Carrier External Review Model Act; Section 9(E)(1) | ERISA | Yes. NAIC Utilization Review and Benefit Determination Model Act; Section 10(B)(1)(a) | ERISA |
| Hawaii |  | HAWAII REV STAT §§ 334B-8 (1991); HAWAII REV. STAT. §§ 432E31 to 432E-44 (2011) |  | Yes. Uniform Health Carrier External Review Model Act; Section 9(E)(1) | ERISA | Yes. NAIC Utilization Review and Benefit Determination Model Act; Section 10(B)(1)(a) | ERISA |
| Idaho | IDAHO CODE ANN. §§ 41-5901 to 41-5917 (2009/2011) | IDAHO CODE ANN §§ 41-5903 to 41-5917 (2009-2011); IDAHO ADMIN. CODE r. 18.04.01.000 to 18.04.01.024 (2019); BULLETIN 2009-8 (2009); BULLETIN 2011-4 (2011). |  | Yes. Uniform Health Carrier External Review Model Act; Section 9(E)(1) | ERISA | Yes. NAIC Utilization Review and Benefit Determination Model Act; Section 10(B)(1)(a) | ERISA |
| Illinois | 215 ILL. COMP. STAT. 180/5 to 180/99 (2010). | 215 ILL COMP STAT 134-85 (2000/2016); 215 ILL. COMP. STAT. 134/45 (2000); ILL. ADMIN. CODE tit. 50, §§ 4530.10 to 4530.90; Exs. A to D (2015); MEMORANDUM 5-27-2010 (2010); BULLETIN 2011-10 (2011). |  | Yes. Uniform Health Carrier External Review Model Act; Section 9(E)(1) | ERISA | Yes. NAIC Utilization Review and Benefit Determination Model Act; Section 10(B)(1)(a) | ERISA |
| Indiana |  | IND CODE §§ 27-8-17-1 to 27-8-17-20 (1992/2015); 760 IND ADMIN CODE §§ 1-46-1 to 1-46-11 (2007/2013); IND. CODE §§ 27-8-29-1 to 27-8-29-24 (2001/2003); §§ 27-13-10.1 to 27-13-10.1-12 (1999/2011) (HMOs); BULLETIN 193 (2012). |  | Yes. Uniform Health Carrier External Review Model Act; Section 9(E)(1) | ERISA | Yes. NAIC Utilization Review and Benefit Determination Model Act; Section 10(B)(1)(a) | ERISA |
| Iowa | IOWA CODE §§ 514J.101 to 514J.120 (2011/2014). | IOWA CODE §§ 514F.1 to 514F.5 (1986-2010); IOWA ADMIN CODE r. 191-70-1 to 191-70.9 (1992/1997); IOWA ADMIN. CODE r. 191-76.1 to 191-76.119 (1999/2012) |  | Yes. Uniform Health Carrier External Review Model Act; Section 9(E)(1) | ERISA | Yes. NAIC Utilization Review and Benefit Determination Model Act; Section 10(B)(1)(a) | ERISA |
| Kansas |  | KAN STAT ANN §§ 40-22a01 to 40-22a13 (1994/2014); KAN ADMIN REGS §§ 40-4-42 to 40-4-42g (1995-2016) |  | Yes. Uniform Health Carrier External Review Model Act; Section 9(E)(1) | ERISA | Yes. NAIC Utilization Review and Benefit Determination Model Act; Section 10(B)(1)(a) | ERISA |
| Kentucky |  | KY REV STAT ANN §§ 304.17A-300 to 304.17A-633 (2000-2017); KY. ADMIN. REGS. 17:290 (2000/2008); BULLETIN 2011-4 (2011) |  | Yes. Uniform Health Carrier External Review Model Act; Section 9(E)(1) | ERISA | Yes. NAIC Utilization Review and Benefit Determination Model Act; Section 10(B)(1)(a) | ERISA |
| Louisiana | LA. REV. STAT. ANN. §§ 22:2431 to 22:2445 (2013) (portions of model). | LA REV STAT ANN §§ 22:2391 to 22:2453 (2013/2017) |  | Yes. Uniform Health Carrier External Review Model Act; Section 9(E)(1) | ERISA | Yes. NAIC Utilization Review and Benefit Determination Model Act; Section 10(B)(1)(a) | ERISA |
| Maine |  | 02-031 ME CODE R ch 850, §§ 1 to 12 (1997/2012) (previous version of model); ME REV STAT ANN tit 24-A, §§ 2342 to 2345 (1989/1995); tit 24-A, §§ 2771 to 2774 (1989/2015); 24-A, § 4304; §4312 (1995/2019); BULLETIN 265 (1997); BULLETIN 397 (2014) |  | Yes. Uniform Health Carrier External Review Model Act; Section 9(E)(1) | ERISA | Yes. NAIC Utilization Review and Benefit Determination Model Act; Section 10(B)(1)(a) | ERISA |
| Maryland |  | MD CODE ANN, INS §§ 15-10B-01 to 15-10B-14 (1998/2016); MD CODE REGS 31.10.21.01 to 31.10.21.12 (1994/2018); 31.10.18.01 to 31.10.18.12 (1999/2005); BULLETIN 2010-30 (2010); BULLETIN 2012-3 (2012). |  | Yes. Uniform Health Carrier External Review Model Act; Section 9(E)(1) | ERISA | Yes. NAIC Utilization Review and Benefit Determination Model Act; Section 10(B)(1)(a) | ERISA |
| Massachusetts |  | 211 MASS CODE REGS 52.01 to 52.18 (2005/2017); MASS. GEN. LAWS ANN. ch. 176O, § 14 (2001/2013); 211 MASS. CODE REGS. 52.03 to 52.16 (2001/2005); 958 MASS. CODE REGS. 3.400 to 3.417 (2014). |  | Yes. Uniform Health Carrier External Review Model Act; Section 9(E)(1) | ERISA | Yes. NAIC Utilization Review and Benefit Determination Model Act; Section 10(B)(1)(a) | ERISA |
| Michigan | NO CURRENT | MICH. COMP. LAWS §§ 550.1901 to 550.1921 (2000/2001). |  | Yes. Uniform Health Carrier External Review Model Act; Section 9(E)(1) | ERISA | Yes. NAIC Utilization Review and Benefit Determination Model Act; Section 10(B)(1)(a) | ERISA |
| Minnesota |  | MINN STAT §§ 62M.01 to 62M.16 (1992/2017); MINN. STAT. § 62Q.73 (2000/2013). |  | Yes. Uniform Health Carrier External Review Model Act; Section 9€(1) | ERISA | Yes. NAIC Utilization Review and Benefit Determination Model Act; Section 10(B)(1)(a) | ERISA |
| Mississippi | 19-1 MISS. CODE R. §§ 15.01 to 15.24 (2012/2014). | MISS CODE ANN §§ 41-83-1 to 41-83-31 (1990/1998) |  | Yes. Uniform Health Carrier External Review Model Act; Section 9(E)(1) | ERISA | Yes. NAIC Utilization Review and Benefit Determination Model Act; Section 10(B)(1)(a) | ERISA |
| Missouri |  | MO REV STAT §§ 376.1350 to 376.1372 (1998/2019) (previous version of model); MO CODE REGS ANN tit 20, § 700-4.100 (1991-2007); §100-5.020; tit 20, §§ 400-10.010 to 400-10.250 (1997-2005) |  | Yes. Uniform Health Carrier External Review Model Act; Section 9€(1) | ERISA | Yes. NAIC Utilization Review and Benefit Determination Model Act; Section 10(B)(1)(a) | ERISA |
| Montana | MONT CODE ANN §§ 33-32-202 to 33-32-217 (1991/2019); §§33-32-401 to 33-32-423 (2015). |  |  | Yes. Uniform Health Carrier External Review Model Act; Section 9(E)(1) | ERISA | Yes. NAIC Utilization Review and Benefit Determination Model Act; Section 10(B)(1)(a) | ERISA |
| Nebraska | NEB. REV. STAT. §§ 44-1301 to 44-1318 (2013/2016). | NEB REV STAT §§ 44-5416 to 44-5431 (1992/1998) (portions of previous version of model); Bulletin CB-123 )2-11_; Bulletin CB-123 (2011). |  | Yes. Uniform Health Carrier External Review Model Act; Section 9(E)(1) | ERISA | Yes. NAIC Utilization Review and Benefit Determination Model Act; Section 10(B)(1)(a) | ERISA |
| Nevada | NEV. REV. STAT. §§ 695G.241 to 695G.310 (2003/2011). | NEV REV STAT §§ 683A.375 to 683A.379 (1991/2015); NEV ADMIN CODE §§ 683A.280 to 683.295 (1992/2010); Bulletin 2011-013 (2011). |  | Yes. Uniform Health Carrier External Review Model Act; Section 9(E)(1) | ERISA | Yes. NAIC Utilization Review and Benefit Determination Model Act; Section 10(B)(1)(a) | ERISA |
| New Hampshire |  | NH REV STAT ANN §§ 420-E:1 to 420-E:9 (1992/2016); NH CODE ADMIN R ANN INS 2001.01 to 2001.19 )1994/2018); 2703.01 to 2703.09 (2011). |  | Yes. Uniform Health Carrier External Review Model Act; Section 9(E)(1) | ERISA | Yes. NAIC Utilization Review and Benefit Determination Model Act; Section 10(B)(1)(a) | ERISA |
| New Jersey |  | N.J. REV. STAT. §§ 26:2S-11 to 26:2S-12 (1997). |  | Yes. Uniform Health Carrier External Review Model Act; Section 9(E)(1) | ERISA | Yes. NAIC Utilization Review and Benefit Determination Model Act; Section 10(B)(1)(a) | ERISA |
| New Mexico |  | NM STAT ANN § 59A-57-4 (1998); N.M. STAT. ANN. § 59A-57-4.1 (2003); N.M. CODE R. §§ 13.10.17.23 to 13.10.17.32 (2000/2004); BULLETIN 2011-012 (2011). |  | Yes. Uniform Health Carrier External Review Model Act; Section 9(E)(1) | ERISA | Yes. NAIC Utilization Review and Benefit Determination Model Act; Section 10(B)(1)(a) | ERISA |
| New York |  | NY INS LAW §§ 4900 to 4908 (1996-2019); NY PUBLIC HEALTH LAW §§ 4900 to 4908 (1996/2019); N.Y. INS. LAW §§ 4910 to 4917 (1999/2014); N.Y. COMP. CODES R. & REGS. tit. 11, §§ 410.1 to 410.13 (2001/2008) (Regulation 166). |  | Yes. Uniform Health Carrier External Review Model Act; Section 9(E)(1) | ERISA | Yes. NAIC Utilization Review and Benefit Determination Model Act; Section 10(B)(1)(a) | ERISA |
| North Carolina | N.C. GEN. STAT. §§ 58-50-75 to 58-50-95 (2002/2014) (portions of model). | NC GEN STAT ANN §§ 58-50-61 to 58-50-62 (1997/2013); Memorandum 8-28-2009. |  | Yes. Uniform Health Carrier External Review Model Act; Section 9(E)(1) | ERISA | Yes. NAIC Utilization Review and Benefit Determination Model Act; Section 10(B)(1)(a) | ERISA |
| North Dakota |  | ND ADMIN CODE §§ 26.1-26.4-01 to 26.1-26.4-05 (1991/2015); BULLETIN 92-1 (1992); N.D. CENT. CODE § 26.1-36-44 (2005/2011); § 26.1-36-46 (2011). |  | Yes. Uniform Health Carrier External Review Model Act; Section 9(E)(1) | ERISA | Yes. NAIC Utilization Review and Benefit Determination Model Act; Section 10(B)(1)(a) | ERISA |
| Ohio | OHIO REV. CODE ANN. §§ 3922.01 to 3922.23 (2011/2012) | OHIO REV CODE ANN §§ 1751-77 to 1751.86 (1997/2019) (previous version of model); OHIO REV CODE ANN § 1753.28 (1997) (emergency services) |  | Yes. Uniform Health Carrier External Review Model Act; Section 9(E)(1) | ERISA | Yes. NAIC Utilization Review and Benefit Determination Model Act; Section 10(B)(1)(a) | ERISA |
| Oklahoma | OKLA. STAT. tit. 63, §§ 6475.1 to 6475.17 (2011/2013) (portions of model). | OKLA STAT tit 36, §§ 6551 to 6565 (1991); OKLA ADMIN CODE §§ 365:10-15-1 to 365:10-15-7 (1997/2010); OKLA. ADMIN. CODE §§ 365:10-29-1 to 365:10-29-10 (2011/2012). |  | Yes. Uniform Health Carrier External Review Model Act; Section 9(E)(1) | ERISA | Yes. NAIC Utilization Review and Benefit Determination Model Act; Section 10(B)(1)(a) | ERISA |
| Oregon |  | OR REV STAT §§ 743B.420 to 743B.425 (1997/2019); OR ADMIN R §§ 836-053-1130 to 836-053-1140 (1998/2013) (small group); OR. REV. STAT. §§ 743b.250 to 743b.258 (2002); OR. ADMIN. R. 836-053-1300 to 836-053-1365 (2002/2014) |  | Yes. Uniform Health Carrier External Review Model Act; Section 9(E)(1) | ERISA | Yes. NAIC Utilization Review and Benefit Determination Model Act; Section 10(B)(1)(a) | ERISA |
| Pennsylvania |  | 40 PA STAT ANN § 991.2152 (1921/1999); 28 PA. CODE § 9.707 (2001); NOTICE 12-31-2011 (2011). |  | Yes. Uniform Health Carrier External Review Model Act; Section 9(E)(1) | ERISA | Yes. NAIC Utilization Review and Benefit Determination Model Act; Section 10(B)(1)(a) | ERISA |
| Rhode Island |  | RI GEN LAWS §§ 27-18.9-1 to 27-18.9-15 (2017/2018) |  | Yes. Uniform Health Carrier External Review Model Act; Section 9(E)(1) | ERISA | Yes. NAIC Utilization Review and Benefit Determination Model Act; Section 10(B)(1)(a) | ERISA |
| South Carolina |  | SC CODE ANN §§ 38-71-1920 to 2060 (2000/2001) (portions of previous version of model); SC CODE ANN §§ 38-70-10 to 38-70-30 (1990/1994); SC CODE ANN REGS 69-47 (1995); Bulletin 4-2011. |  | Yes. Uniform Health Carrier External Review Model Act; Section 9€(1) | ERISA | Yes. NAIC Utilization Review and Benefit Determination Model Act; Section 10(B)(1)(a) | ERISA |
| South Dakota |  | SD CODIFIED LAWS §§ 58-17H-1 to 58-17H-52 (2011/2015) (portions of previous version of model); SD AMDIN R 20:06:33; 20:06:53 (1999/2013) |  | Yes. Uniform Health Carrier External Review Model Act; Section 9(E)(1) | ERISA | Yes. NAIC Utilization Review and Benefit Determination Model Act; Section 10(B)(1)(a) | ERISA |
| Tennessee |  | TENN CODE ANN §§ 56-6-701 to 56-6-706 (1992/2014); TENN. CODE ANN. §§ 56-61-101 to 56-61-125 (2010). |  | Yes. Uniform Health Carrier External Review Model Act; Section 9(E)(1) | ERISA | Yes. NAIC Utilization Review and Benefit Determination Model Act; Section 10(B)(1)(a) | ERISA |
| Texas |  | 28 TEX ADMIN CODE §§ 19-1701 to 19.1719 )2013/2019); TEX. INS. CODE ANN. §§ 4201.401 to 4201.403 (2007/2009); 28 TEX. ADMIN. CODE §§ 19.1701 to 19.1719 (2013); BULLETIN B-0051-11 (2011). | HHS | Yes. Uniform Health Carrier External Review Model Act; Section 9(E)(1) | ERISA | Yes. NAIC Utilization Review and Benefit Determination Model Act; Section 10(B)(1)(a) | ERISA |
| Utah |  | UTAH ADMIN. CODE r. 590-203-1 to 590-203-10 (2002/2011). |  | Yes. Uniform Health Carrier External Review Model Act; Section 9(E)(1) | ERISA | Yes. NAIC Utilization Review and Benefit Determination Model Act; Section 10(B)(1)(a) | ERISA |
| Vermont |  | VT. STAT. ANN. tit. 8, § 4089f (1997/2011); VT. ADMIN. CODE §§ 4-5-4:1 to 4-5-4:13 (Rule H-2011-02) (2011). |  | Yes. Uniform Health Carrier External Review Model Act; Section 9(E)(1) | ERISA | Yes. NAIC Utilization Review and Benefit Determination Model Act; Section 10(B)(1)(a) | ERISA |
| Virginia |  | VA CODE ANN §§ 32.1-137.7 to 32.1-137.17 (1998/2011); 14 VA. ADMIN. CODE §§ 5-216-10 to 5-216-130 (2011/2012); VA. CODE ANN. §§ 32.1-137.7 to 32.1-137.17 (1998/2011) |  | Yes. Uniform Health Carrier External Review Model Act; Section 9(E)(1) | ERISA | Yes. NAIC Utilization Review and Benefit Determination Model Act; Section 10(B)(1)(a) | ERISA |
| Washington |  | WASH ADMIN CODE §§ 284-43-3000 to 284-43-3190 (2011/2016); WASH. REV. CODE § 48.43.535 (2011); WASH. ADMIN. CODE. § 284-43A-140 (2016); §§ 284-43-3000 to 284-43-3190 (2016); §§ 284-43-4000 to 4040 (2015). |  | Yes. Uniform Health Carrier External Review Model Act; Section 9(E)(1) | ERISA | Yes. NAIC Utilization Review and Benefit Determination Model Act; Section 10(B)(1)(a) | ERISA |
| West Virginia | W. VA. CODE R. §§ 114-97-1 to 114-97-15 (2014). |  |  | Yes. Uniform Health Carrier External Review Model Act; Section 9(E)(1) | ERISA | Yes. NAIC Utilization Review and Benefit Determination Model Act; Section 10(B)(1)(a) | ERISA |
| Wisconsin |  | WIS. STAT. § 632.835 (2000); WIS. ADMIN. CODE INS. §§ 18.10 to 18.18 (2001/2012) | HHS | Yes. Uniform Health Carrier External Review Model Act; Section 9(E)(1) | ERISA | Yes. NAIC Utilization Review and Benefit Determination Model Act; Section 10(B)(1)(a) | ERISA |
| Wyoming |  | W.S. 26-40-201: WY. Ins. Reg. Ch. 63 (2011) |  | Yes. Uniform Health Carrier External Review Model Act; Section 9(E)(1) | ERISA | Yes. NAIC Utilization Review and Benefit Determination Model Act; Section 10(B)(1)(a) | ERISA |
| Abbreviations: IRO, independent review organization; NAIC, National Association of Insurance Companies; HHS, United State Department of Health and Human Services; ERISA, Employee Retirement Income Security Act | | | | | | | |
